# Unveiling the molecular mechanism of sepal curvature in *Dendrobium* Section *Spatulata* through full-length transcriptome and RNA-seq analysis

**DOI:** 10.3389/fpls.2024.1497230

**Published:** 2024-12-13

**Authors:** Xuefeng Qu, Na Li, Cong Xu, Zifeng Huang, Chunyan Li, Yang Jiang, Guizhao Zheng, Haiping Fu, Guangyan Zhang, Chuan Liu

**Affiliations:** ^1^ Dongguan Research Center of Agricultural Sciences, Dongguan, Guangdong, China; ^2^ Chongqing Key Laboratory of Big Data for Bio Intelligence, Chongqing University of Posts and Telecommunications, Chongqing, China

**Keywords:** *Dendrobium*, floral development, sepal curvature, transcriptomics, cytokinesis, myosin filament

## Abstract

**Introduction:**

Orchids are renowned for their intricate floral structures, where sepals and petals contribute significantly to ornamental value and pollinator attraction. In *Dendrobium* Section *Spatulata*, the distinctive curvature of these floral organs enhances both aesthetic appeal and pollination efficiency. However, the molecular and cellular mechanisms underlying this trait remain poorly understood.

**Methods:**

Morphological characteristics of five hybrids were analyzed, with a particular focus on hybrid H5, which exhibits pronounced sepal curling. Full-length transcriptomic sequencing was employed to assemble a reference transcriptome, while RNA-seq identified differentially expressed genes (DEGs) between sepals and petals. Gene ontology and pathway enrichment analyses were conducted to uncover biological processes associated with sepal curvature. Cytological microscopy was used to examine cell size and number, and quantitative real-time PCR (qRT-PCR) was performed to validate transcriptomic findings.

**Results:**

The reference transcriptome contained 94,258 non-redundant transcripts, and RNA-seq identified 821 DEGs between sepals and petals, with 72.8% of these upregulated in sepals. Enrichment analysis revealed the significant involvement of DEGs in cytokinesis, cytoskeletal organization, and energy metabolism. Notably, myosin II filament organization was implicated in generating the mechanical forces responsible for curling, while metabolic pathways provided the energy necessary for these developmental processes. Cytological observations showed that the upper cell layers of the sepal were smaller and more numerous than the lower layers, indicating that differential cell growth contributes to sepal curvature. qRT-PCR analysis validated the differential expression of selected genes, supporting the transcriptomic findings.

**Discussion:**

The interplay of cellular mechanics, cytoskeletal dynamics, and metabolic regulation is crucial in shaping sepal morphology. Future studies involving gene knockdown or overexpression experiments are recommended to validate the roles of specific genes in processes such as actin organization and myosin activity. Such work would provide deeper insights into the contributions of cytoskeletal dynamics and mechanical force generation to sepal morphogenesis.

## 1 Introduction

Orchids, a highly diverse and economically significant plant family, are renowned for their intricate and striking floral structures. Among the various floral organs, the shape, size, and color of sepals and petals are crucial factors influencing both their ornamental value and their attractiveness to pollinators. Within the Orchidaceae family, *Dendrobium*, a genus prized for its vibrant and attractive flowers, holds significant economic importance in the cut-flower and potted plant industries (Ketsa and Warrington, 2023). *Dendrobium* species are characterized by diverse floral forms, and *Dendrobium* Section *Spatulata* (*Den.* Sect*. Spatulata*), in particular, is noted for the distinctive curvature of its sepals and petals. This curvature not only contributes to the aesthetic appeal of the flowers but may also play a role in pollination efficiency, potentially enhancing the reproductive success of these species (Moyroud and Glover, 2017; Khojayori et al., 2024). Furthermore, sepal curvature could serve as an important phenotypic trait in ornamental breeding, helping to distinguish between different *Dendrobium* varieties and hybrids. This could be invaluable for classification and the development of new hybrid strains with desirable floral characteristics.

Despite the economic and ornamental significance of floral morphology in *Dendrobium*, the genetic and developmental mechanisms governing sepal and petal curvature remain poorly understood. Floral development in *Dendrobium* follows the conserved Perianth (P) code model, where protein complexes composed of AP3/AGL6 homologs regulate the formation of sepals, petals, and lips (Hsu et al., 2015). Recent studies have suggested that the regulation of floral organogenesis in *Dendrobium* may involve a more complex interplay of MADS-box genes and hormonal pathways (Wang et al., 2020; Zhang et al., 2022). However, the specific molecular and cellular mechanisms underlying the curvature of sepals and petals in *Dendrobium* have not been thoroughly explored.

The central aim of this study is to investigate the molecular and cellular basis of sepal curvature in *Den.* Sect*. Spatulata*. We focus on a particular hybrid, H5, which exhibits pronounced sepal curling while maintaining relatively flat petals. This unique phenotype provides an ideal model to examine the mechanisms driving sepal curvature independent of petal morphology. Our primary research questions are as follows: (1) What are the key genes and pathways involved in regulating sepal curvature in *Den.* Sect*. Spatulata*? (2) How do cellular and metabolic processes contribute to the morphological changes observed in the sepal? We hypothesize that differential gene expression related to cytoskeletal dynamics, cell growth, and metabolic regulation plays a critical role in driving sepal curvature in this hybrid. Through transcriptomic analysis of hybrid H5, we aim to uncover the molecular mechanisms underlying sepal curvature and, by extension, deepen our understanding of floral organ development in orchids. Furthermore, this study provides a broader contribution to plant developmental biology by offering insights into the regulation of curvature in floral organs and their potential applications in ornamental horticulture.

## 2 Materials and methods

### 2.1 Plant materials and growth conditions

Five hybrids from *Den.* Sect*. Spatulata*, designated H1–H5, were used to assess floral organ phenotypes. These hybrids were initially acquired from orchid markets or local farms. Hybrids H1–H4 likely originated from genetic crosses involving *Den. stratiotes*, *Den. lasianthera*, *Den.* Panavee, and other varieties. Hybrid H5, also from *Den.* Sect*. Spatulata*, has an untraceable parentage and was initially obtained from an orchid farm in Hainan Province, China. All the *Dendrobium* plants were subsequently cultivated in our greenhouse. Based on greenhouse observations, it takes approximately 25 days for flower buds to develop from their initial separation from clustered growth to blooming. During this period, flower bud length increases from an average of 9.5 mm to 30 mm. Consequently, the developmental stages of *Den.* Sect*. Spatulata* were classified as follows: Stage 1, buds shorter than 16.00 mm after separation from clustered growth; Stage 2, buds between 16.00 mm and 23.00 mm; Stage 3, buds exceeding 23.00 mm; Stage 4, blooming initiation. The observation of plant growth and flowering, along with RNA-seq sampling, began in August 2022. For each developmental stage, four replicates of petals and sepals were collected, with approximately 1g of tissue per replicate. Three replicates were used for Illumina short-read sequencing, while the fourth replicate was pooled and used for long-read sequencing. All samples were immediately frozen in liquid nitrogen and stored at − 80 °C.

### 2.2 RNA extraction and detection

Total RNA from the sepals and petals of *Den.* Sect*. Spatulata* was extracted using Tiangen DP411 (Tiangen, Beijing, China) according to the manufacturer’s instructions. RNA concentration and integrity were analyzed using a NanoDrop 2000 spectrophotometer (Thermo, MA, USA) and an Agilent 2100, LabChip GX (Perkin Elmer, Shanghai, China). Qualified RNA was stored at -80°C for later use.

### 2.3 Illumina short-read library construction and sequencing

A total of 1 μg of RNA per sample was used as the input material for library preparation. The mRNA was purified from the total RNA using HieffNGS DNA selection Beads (Yeasen, Shanghai, China). Sequencing libraries were generated from the purified mRNA using the Hieff NGS Ultima Dual-mode mRNA Library Prep Kit for Illumina (Yeasen, Shanghai, China) following the manufacturer’s recommendations with unique index codes. The library quantification and size were assessed using a Qubit 3.0 fluorometer (Life Technologies, CA, USA) and an Agilent 2100, LabChip GX (Perkin Elmer, Shanghai, China). Subsequently, sequencing with a paired‐end sequencing length of 150 bp (PE150) was performed on the Illumina Novaseq 6000 platform (Illumina, CA, USA) by Biomarker Technologies Co., Ltd. (Beijing, China). The raw sequencing data have been deposited in the NCBI Sequence Read Archive (SRA) under BioProject accession number PRJNA1129577.

### 2.4 PacBio long-read library generation and sequencing

A total of 600 ng RNA was used to generate the PacBio long-read library. Full-length (FL) cDNA was synthesized using the NEBNext Single Cell/Low Input cDNA Synthesis and Amplification Module (New England Biolabs, MA, USA) and subsequently amplified. Size selection of the FL cDNA (1–6 kb) was performed using the BluePippin system (Sage Science, MA, USA). The ends of the FL cDNA were repaired and the hairpin sequencing adapters were ligated using the SMRTbell^®^ Express Template Prep Kit 2.0 (Pacific Biosciences, CA, USA). Library quality and quantity were evaluated using an Agilent 2100, LabChip GX (Perkin Elmer, Shanghai, China) and a Qubit 3.0 fluorometer (Life Technologies, CA, USA). The polymerase-bound SMRTbell libraries were sequenced on the Pacific Biosciences Sequel II System (Pacific Biosciences, CA, USA). The raw sequencing data have been deposited in the NCBI SRA under BioProject accession number PRJNA1129577.

### 2.5 PacBio long-read processing

The raw subreads were analyzed following the Iso-Seq3 pipeline (https://github.com/PacificBiosciences/IsoSeq). The pipeline included three initial steps: generation of the circular consensus sequence (CCS) subreads, classification of the FL reads, and clustering of the full-length non-chimeric (FLNC) reads. Polished CCS subreads were generated, using CCS v6.2.0, from the subreads bam files with a minimum quality of 0.9 (–min-rq 0.9). The default minimum number of FL subreads (n = 3) required to generate CCS for a zero-mode waveguide (ZMW) was used. FL transcripts were determined when the sequences had the poly(A) and the 5′ and 3′ cDNA primers. Lima v2.1.0 and isoseq3 refine were used to remove the primers and poly(A) tails, respectively. The clustering algorithm ICE was used to obtain high-quality FL consensus sequences. High-quality FL consensus sequences were classified with the criterion of a post-correction accuracy above 0.99. High-quality full-length (FL) transcripts obtained through Iso-Seq were processed using CD-HIT with an identity threshold greater than 0.99 to eliminate redundancy (Fu et al., 2012).

### 2.6 Gene structure analysis

Simple sequence repeats (SSRs) of the transcriptome were identified using MISA (Beier et al., 2017). TransDecoder (https://github.com/TransDecoder/TransDecoder/releases) was used to identify candidate coding regions within transcript sequences based on several criteria. First, a minimum-length open reading frame (ORF) must be present in the transcript sequence. Second, the log-likelihood score, similar to the score computed by GeneID, must be greater than 0. Third, the coding score should be highest when the ORF is scored in the first reading frame compared to the other five frames. Additionally, if one ORF is fully encapsulated by the coordinates of another, the longer ORF is reported, though multiple ORFs may be reported per transcript to account for operons, chimeras, and other scenarios. Optionally, the putative peptide may match a Pfam domain with a score above the noise cutoff. Iso-Seq™ data were used to perform an all-vs-all BLAST with high-identity settings, and alignments that met the following criteria were considered products of candidate alternative splicing (AS) events: the alignment must contain two High-Scoring Segment Pairs (HSPs) in the same forward or reverse direction, one sequence must be continuous or have a small overlap (less than 5 bp), and the other sequence must exhibit a distinct “AS Gap”. The continuous sequence should nearly fully align with the distinct sequence, and the AS Gap must be greater than 100 bp and located at least 100 bp away from the 3’ or 5’ ends.

### 2.7 RNA-seq data processing

Raw sequencing data (in fastq format) were initially processed using in-house Perl scripts. During this step, clean reads were obtained by filtering out reads containing adapters, poly-N sequences, and low-quality reads. Concurrently, various quality metrics, including Q20, Q30, GC-content, and sequence duplication levels, were calculated for the clean data. All subsequent analyses were performed using high-quality clean data.

### 2.8 Quantification of gene expression levels and differential expression analysis

The PacBio sequencing data generated in this study served as the reference transcriptome. Kallisto software was used to construct an index file (Kallisto index transcriptome.fa -i transcriptome.indx), followed by the quantification of gene expression levels for each sample (Kallisto quant -i transcriptome.index -o outfile R1.fastq.gz R2.fastq.gz). Gene expression levels were estimated using the number of transcripts per kilobase million (TPM) mapped. Differentially expressed genes (DEGs) were identified using the DESeq2 package (Love et al., 2014). Gene Ontology (GO) enrichment analysis was conducted with GO annotation data using the clusterProfiler 4.0 package (The Gene Ontology Consortium, 2019; Wu et al., 2021). Kyoto Encyclopedia of Genes and Genomes (KEGG) pathway enrichment analysis was performed using KOBAS 3.0 (Bu et al., 2021).

### 2.9 qRT-PCR and cytological microscopy

To validate gene expression, total RNA was extracted from petal and sepal samples collected at the same developmental stages as those used for the transcriptome sequencing. RNA extraction was performed using the Total RNA Extraction Kit for polyphenol- and polysaccharide-rich plants (R4150; Magen Biotech, Guangzhou, China). Complementary DNA (cDNA) was synthesized from the extracted RNA using the Hifair^®^ II 1st Strand cDNA Synthesis Kit (11119ES60; Yeasen Biotech, Shanghai, China). Quantitative real-time PCR (qRT-PCR) was conducted with Hieff^®^ qPCR SYBR Green Master Mix (Low Rox Plus) (11202ES08; Yeasen Biotech, Shanghai, China). The primer sequences used for gene expression validation are provided in **Supplementary Table S1**.

The samples were organized into dehydration cassettes and processed through a series of ethanol solutions: 75% for 4 hours, 85% for 2 hours, 90% for 2 hours, and 95% for 2 hours, followed by two rounds of absolute ethanol for 1 hour each. They were then immersed in an ethanol-xylene mixture for 5-10 minutes, followed by two changes of xylene for 5-10 minutes each. Paraffin infiltration involved treating the samples in a paraffin-xylene mixture at 62°C for 2 hours and two changes of melted paraffin at 62°C for 4 hours each. The paraffin-infiltrated tissues were transferred to an embedding station, placed into molds with melted paraffin, and cooled on a -20°C plate. The solidified paraffin blocks were trimmed, sectioned into 8-10 µm slices using a microtome, and floated on a 40°C water bath before being mounted on slides and baked at 60°C. The sections were deparaffinized in xylene and rehydrated through a series of ethanol solutions before toluidine blue staining for 2-5 minutes. Finally, the sections were rendered transparent by immersion in clean xylene, mounted with neutral balsam, and observed under a microscope.

## 3 Results

### 3.1 Morphological analysis of flower organs in *Den.* Sect*. Spatulata*

**Figure 1:**
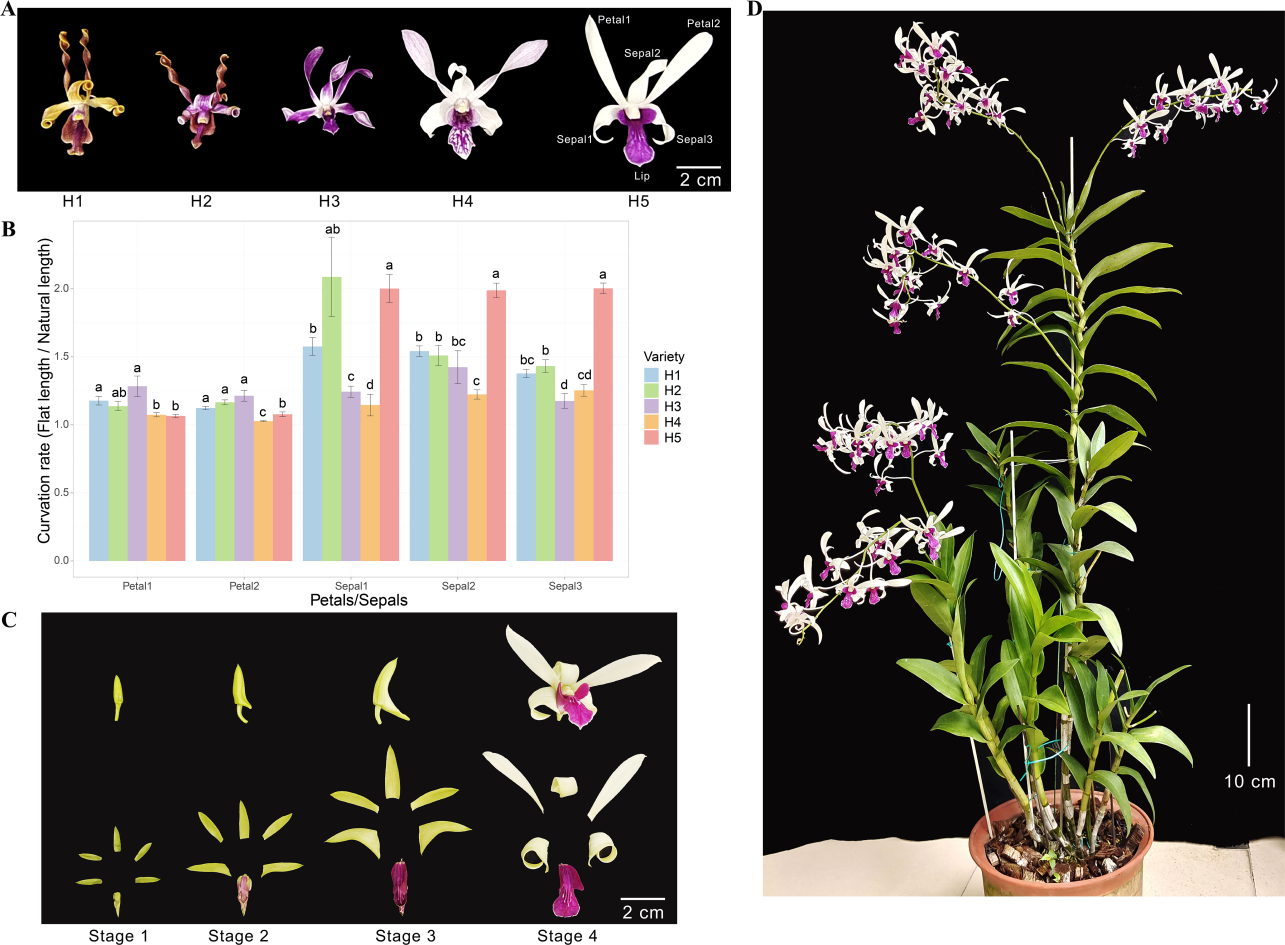
Morphological characterizations of plant and flower organs. **(A)** Morphological phenotypes of flowers from five orchid hybrids (H1, H2, H3, H4, and H5) from *Den.* Sect. *Spatulata*. The petals and sepals used for the curvation rate calculations were numbered and indicated on H5. **(B)** Comparison of the curvation rates for petals and sepals among the five hybrids. The curvation rate was determined by dividing the natural length by the flat length of the petals or sepals. Significant differences between hybrids for each organ were assessed using multiple mean comparisons. Different letters above the bars within each organ indicate statistical significance (*P* < 0.05, *t*-test). **(C)** Morphological phenotypes of H5 flower organs at various flowering stages. **(D)** Image of the H5 plant at the late flowering stage.

*Den.* Sect*. Spatulata* usually show spirally twisted floral segments and asymmetrical flowers, resembling antelope horns. **Figure 1A** shows the flowers of five hybrids (H1-H5) from *Den.* Sect*. Spatulata* that were preserved in our germplasm resources. All these hybrids possess curled sepals. The petals of H1 and H2 were curled up into typical spirals, and those of H3 and H4 were gently curled up. However, H5 exhibited contrasting flower organs as the sepal was curled up, whereas the petal remained almost flat (**Figure 1A**). The natural and flat lengths of petals and sepals were measured, and the curvation rate was obtained by dividing flat length by natural length. Except for H3, all the sepals had roughly higher curvation rates than the petals (**Figure 1B**). The curvation rate of the H5 sepals was substantially higher than that of the petals and was also higher than most of the sepals in the other four hybrids. As can be seen from the four developing stages of H5 flower organs, the sepals were curled the most in Stage 4 (**Figure 1C**). The plant height of H5 exceeded 1.2 m (**Figure 1D**), and its biomass supported a higher annual booming frequency. These characteristics make H5 of *Den.* Sect*. Spatulata* an ideal plant for studying the molecular mechanism of sepal curvature.

### 3.2 Full-length transcriptome sequencing and quality assessment

**Figure 2:**
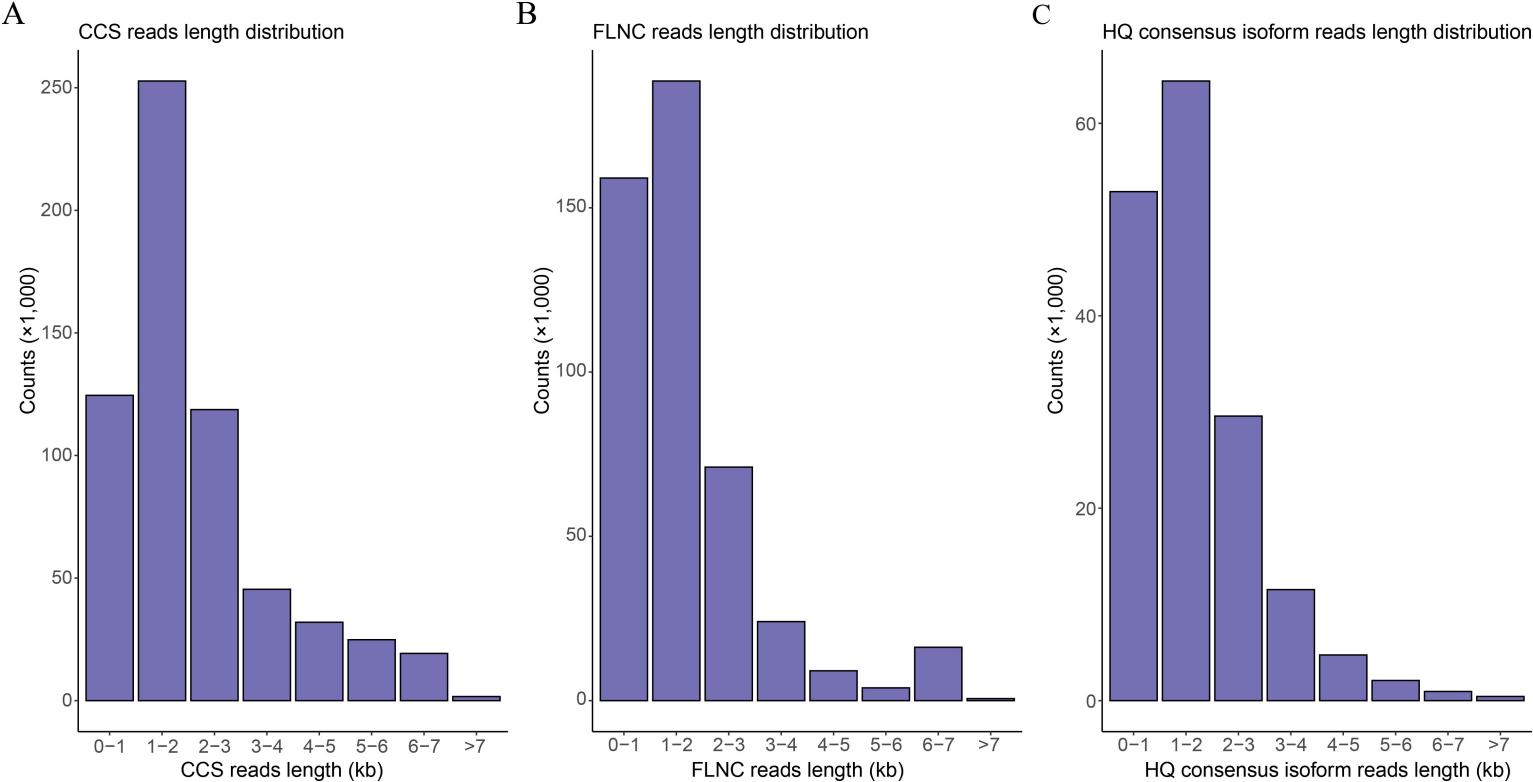
Read length distribution of the full-length transcriptome sequences. **(A)** Distribution of the CCS read lengths. **(B)** Distribution of the FLNC read lengths. **(C)** Distribution of the read lengths for the high-quality (HQ) consensus isoforms.

Full-length transcriptome sequencing of H5 in *Den.* Sect*. Spatulata* generated a total of 62.62 Gb of data, in which 619,157 CCS reads were obtained. The distribution of CCS read lengths shows that the number of CCS reads was 124,515 for 0-1 kb, 252,760 for 1-2 kb, 118,699 for 2-3 kb, 45,406 for 3-4 kb, and 77,776 for >4 kb (**Figure 2A**; **Supplementary Table S2**). Further, a total of 472,576 FLNC reads were obtained by filtering adaptor and low-quality sequences, accounting for 76.33% of the CCS reads. The number of FLNC reads from the library was 159,049 for 0-1 kb, 188,585 for 1-2 kb, 71,077 for 2-3 kb, 24,050 for 3-4 kb, and 29,864 for >4 kb, respectively (**Figure 2B**; **Supplementary Table S2**). Since the FLNC reads in the cDNA library contained duplicate isoforms, the similar FLNC read sequences were clustered by the IsoSeq module in SMRTLink. This step generated 166,706 consensus isoforms, containing 166,650 high-quality (HQ) consensus isoforms with an accuracy higher than 0.99. The average lengths of HQ consensus isoform reads were 632, 1,461, 2,421, 3,425 and 5,090 bp for the 0-1 kb, 1-2 kb, 2-3 kb, 3-4 kb and >4 kb libraries, respectively (**Figure 2C**; **Supplementary Table S2**). Finally, a total of 94,258 non-redundant transcripts were obtained by removing the redundant transcripts with the CD-HIT software using the clustering threshold of “-c 0.99”.

**Figure 3:**
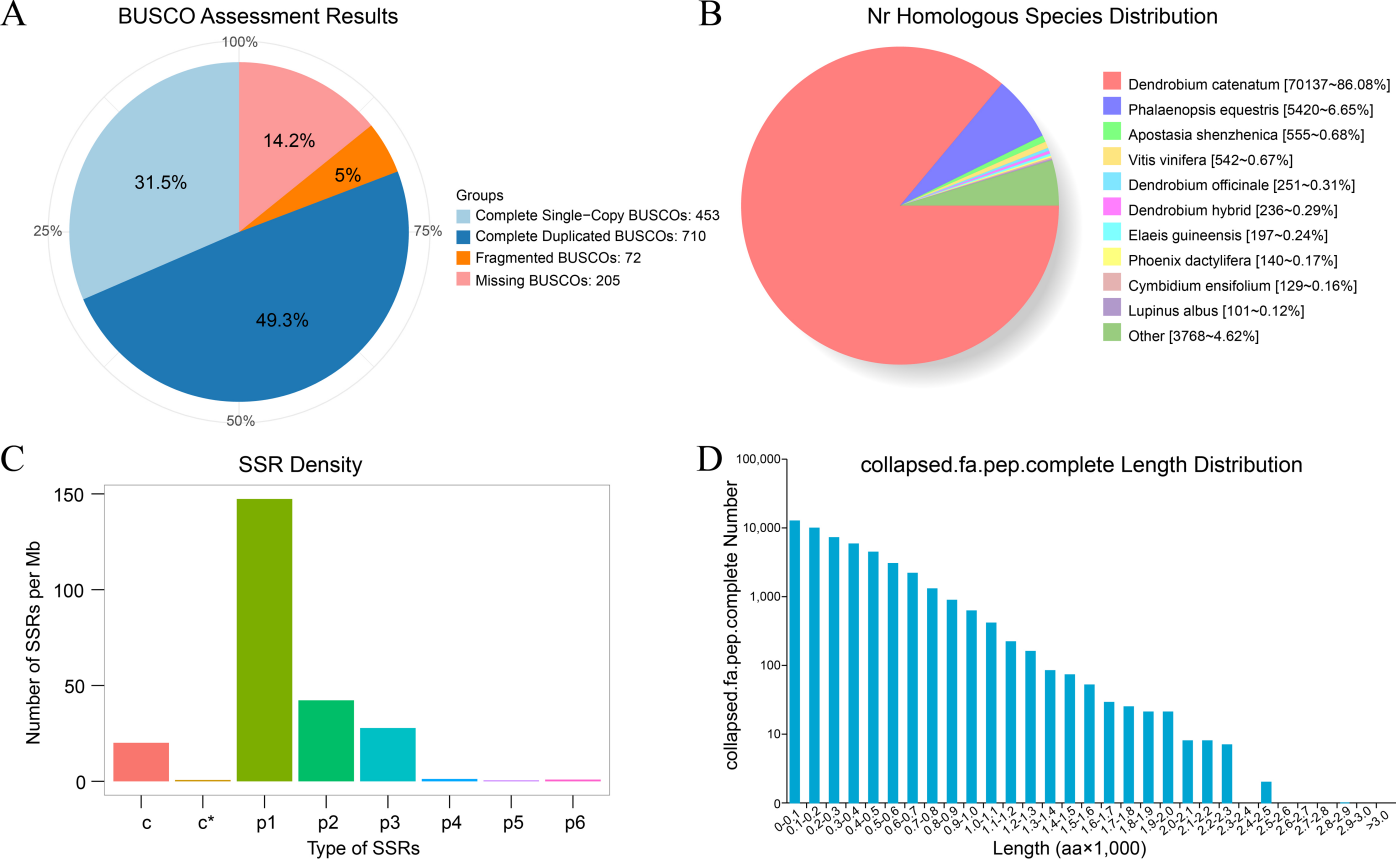
Analysis of transcripts and proteins derived from the full-length transcriptome. **(A)** Transcript completeness assessment using BUSCO software. **(B)** Distribution of nr homologous species annotated with non-redundant transcript sequences. **(C)** SSR density for eight types of predicted SSRs, identified using MISA software. **(D)** Length distribution of the predicted complete proteins.

After removing the redundant transcripts, BUSCO (version 3.0.2) was used to assess the completeness of the non-redundant transcripts. Among the total of 1,440 BUSCO groups, 80.8% (1,163) were complete BUSCOs, including 453 single-copy BUSCOs and 710 duplicated BUSCOs (**Figure 3A**). These results indicated that most of the non-redundant transcripts were assembled with high quality. Alternative splicing is crucial for generating transcripts with different combinations of exons, which can thus be translated to different proteins and result in biological diversity. To identify the potential alternative splicing events, all the non-redundant transcripts were used to perform pairwise sequence alignment using the criteria described in the methods section. Finally, a total of 2,762 potential alternative splicing events were obtained (**Supplementary Table S3**). These data may support further studies focusing on the identification of robust alternative splicing events.

### 3.3 Functional annotation, SSR analysis, and coding sequence prediction

In order to obtain annotation information for the transcriptome, all the non-redundant transcripts (94,258) were aligned to nucleotide or protein databases. A total of 82,254 transcripts were finally annotated, of which 66,964, 57,071, 23,660, 47,497, 57,628, 55,649, 81,586, 69,340, and 81,477 transcripts were annotated in the GO, KEGG, COG, KOG, Pfam, Swiss-Prot, TrEMBL, eggNOG and nr databases, respectively (**Supplementary Table S4**). The nr homologous species distribution showed that 86.08% of the nr annotated sequence was consistent with the published sequences of *Dendrobium catenatum*, and the second had 6.65% sequence identity with *Phalaenopsis equestris* (**Figure 3B**). These results suggested that the full-length transcriptome sequencing obtained robust data and can be used as a reference transcriptome in the downstream analysis.

SSR is widely applied in the construction of a genetic linkage map and analysis of genetic diversity in orchids. MISA software was used to identify SSR from the transcripts with over 500 bp. A total of 39,157 SSR sites were obtained, including 23,961 mono nucleotides (p1), 6,871 di nucleotides (p2), 4,519 tri nucleotides (p3), 199 tetra nucleotides (p4), 85 penta nucleotides (p5), 145 hexa nucleotides (p6), 3,263 compound SSRs (c), and 114 compound SSRs with overlapping positions (c*) (**Supplementary Table S5**). The top three high-density SSR sites were p1, p2, and p3 with 147.35, 42.25, and 27.79 SSR sites per Mb, respectively (**Figure 3C**). The coding sequence and protein sequence of all the transcripts were predicted using TransDecoder software (v5.0.0). A total of 81,862 ORFs were obtained, of which 49,209 (60.11%) were complete. The number of proteins with complete ORFs was the highest at the length of 0-100 amino acids, and gradually decreases as the length of protein increases (**Figure 3D**).

### 3.4 RNA-seq data assessment and identification of DEGs between sepals and petals

**Figure 4:**
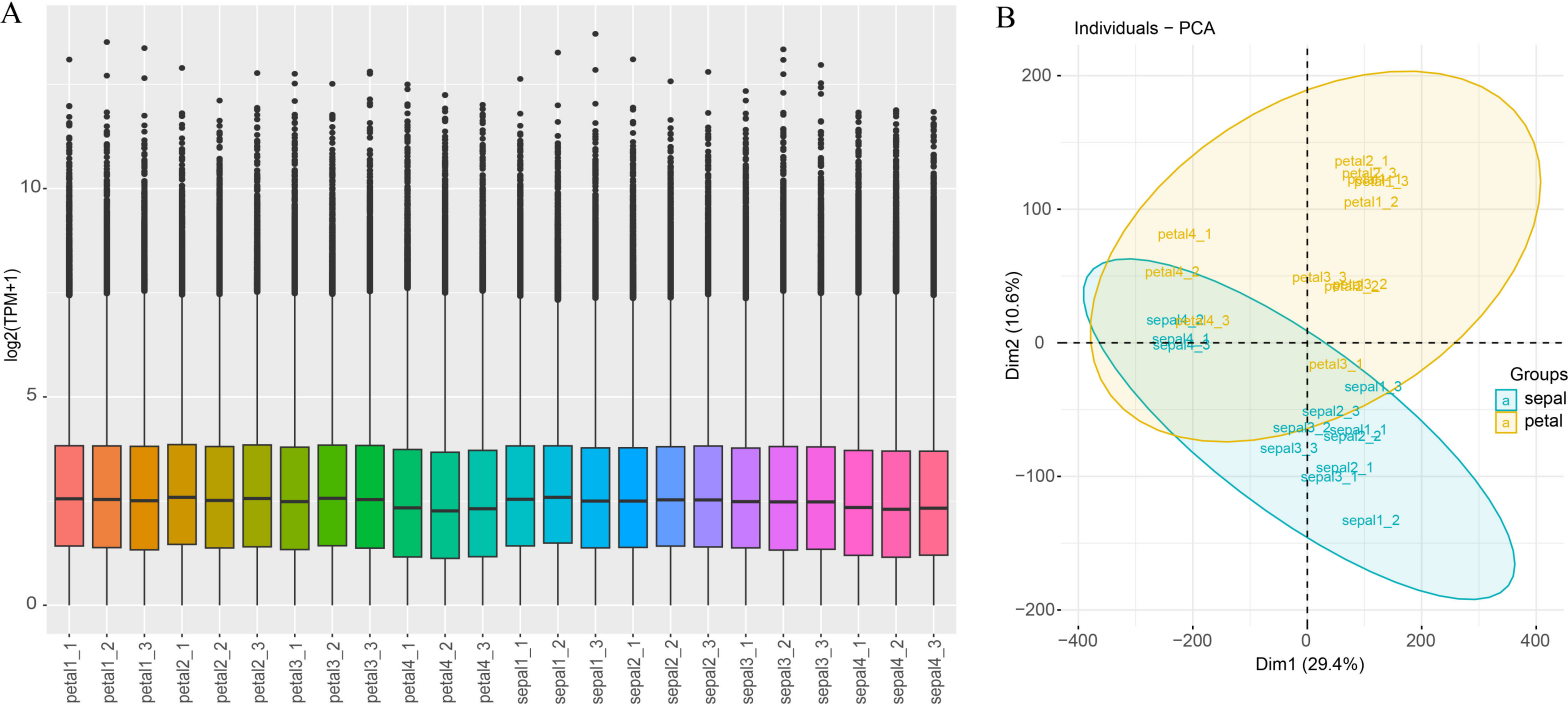
Gene expression distribution and PCA analysis of all samples. **(A)** Expression distribution across all samples. The x-axis represents the 24 samples used in this study, and the y-axis represents gene expression normalized by log_2_(TPM+1). **(B)** Principal component analysis (PCA) of all the samples, with sepals and petals represented by blue and yellow, respectively.

Illumina high-throughput RNA sequencing (RNA-seq) was used to quantify the gene expression levels of 12 sepal samples and 12 petal samples, both contain 3 replicates of 4 developing stages of H5 flower organs. Generally, the number of clean reads ranged between 20,360,805 (petal1-1) and 26,910,018 (petal4-2), with an average of 22,982,401. The percentages of Q30 ranged between 92.78% and 94.09%, with an average of 92.78% (**Supplementary Table S6**). Of these clean read pairs, 45.54% to 48.13% were uniquely mapped to the reference transcriptome, and 40.34% to 43.96 were mapped to multiple loci (**Supplementary Table S6**). Both the uniquely mapped reads and the reads mapped to multiple loci were counted by Kallisto (v0.50.0) software and the counts were normalized to TPM. The TPM value of each sample was used to assess the total expression status and sample correlations. Most of the samples showed a similar expression distribution pattern (**Figure 4A**). Only the TPM value of the samples in Stage 4 (petal4_1 to petal4_3 and sepal4_1 to sepal4_3) was slightly lower than other samples, possibly due to the phenotypic difference between Stage 4 and other stages. This result was consistent with the principal component analysis (PCA) which revealed a general difference between petal and sepal samples, and the Stage 4 samples obviously clustered together (**Figure 4B**). Meanwhile, the sample correlation analysis showed a higher correlation coefficient between the replicated samples than that between non-replicated samples (**Supplementary Figure S1**). These results demonstrate that the RNA-seq data were sufficient and reliable for subsequent differential expression analysis.

**Figure 5:**
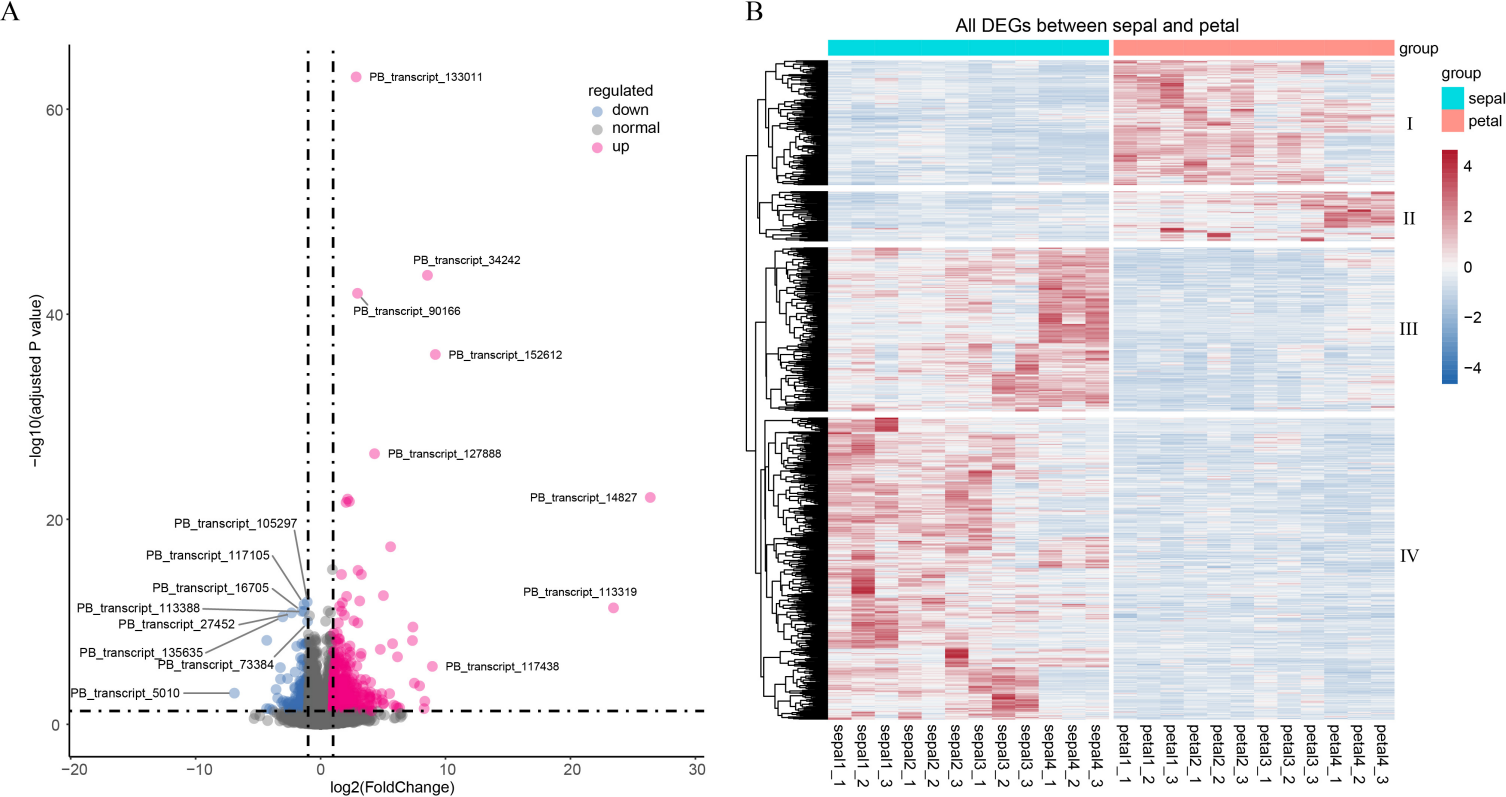
Volcano plot and heatmap of differentially expressed genes (DEGs). **(A)** Volcano plot showing downregulated (blue), normal (grey), and upregulated (red) genes. **(B)** Heatmap of 223 downregulated and 598 upregulated DEGs. TPM values were scaled by row using the “pheatmap” package in R. Sepal and petal samples are indicated by the cyan and red horizontal bars above the heatmap. The four gene clusters are marked on the right side of the heatmap.

Although the curling phenotype was most prominent in Stage 4, we hypothesize that this trait is the culmination of regulatory events driven by specific genes across the floral developmental stages. To identify the underlying genes involved in sepal curvature, we conducted a bulk analysis by comparing sepals and petals across 12 samples from the four stages. This analysis revealed a total of 821 DEGs between sepals and petals. Notably, 598 DEGs (72.8%) were upregulated, while 223 DEGs (27.2%) were downregulated (**Figure 5A**). These DEGs displayed four distinct expression clusters: downregulated DEGs were grouped into clusters I and II, while upregulated DEGs formed clusters III and IV (**Figure 5B**). Gene cluster I in petals and cluster IV in sepals showed higher expression during the early stages of flower organ development (Stages 1 to 3). In contrast, gene cluster II in petals and gene cluster III in sepals peaked at Stage 4. Particularly, gene cluster III in sepals demonstrated a gradual increase in expression from early to late stages, suggesting a significant role for these DEGs in sepal morphogenesis.

### 3.5 Functional enrichment of DEGs reveals roles in cytokinesis and metabolic pathways during sepal development

**Figure 6:**
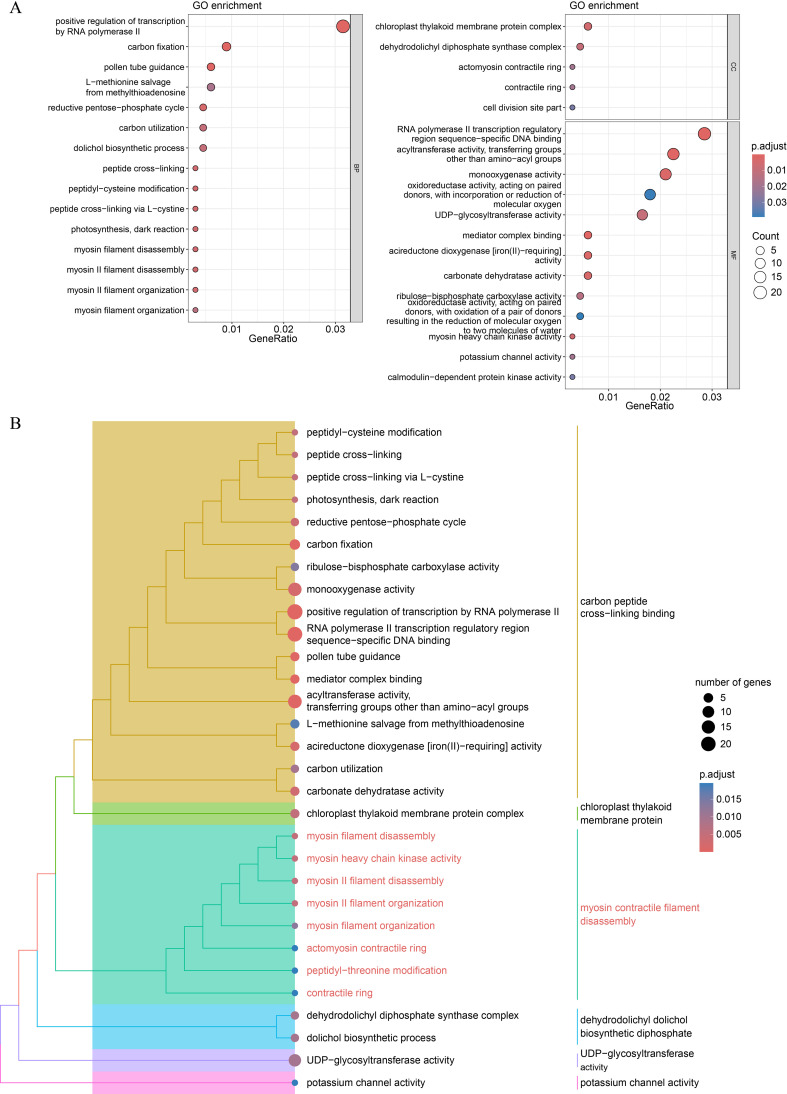
GO functional enrichment analysis of the DEGs. **(A)** Dot plot of the significantly enriched GO terms for all the DEGs. **(B)** Hierarchical tree of significant GO terms. The GO terms with an adjusted *P*-value < 0.05 were considered significant. For categories with more than 15 significant terms, only the top 15 terms in each category (BP, Biological Process; CC, Cellular Component; MF, Molecular Function) were plotted.

GO and KEGG pathway enrichment analyses were conducted to examine the functional characteristics of the DEGs. Several GO terms were significantly enriched, including positive regulation of transcription by RNA polymerase II, carbon fixation, and pollen tube guidance (**Figure 6A**; **Supplementary Table S7**). Semantic similarity clustering was applied to group-related GO terms. We identified two subtrees with key terms including carbon peptide cross-linking binding and myosin contractile filament disassembly, which contained the majority of the enriched GO terms (**Figure 6B**). Peptide cross-linking, myosin II filament organization, and actomyosin contractile ring were frequently enriched in these two subtrees. Myosin II filaments play a critical role in regulating both the length and alignment of actin filaments in the contractile rings during cell division (Arima et al., 2023). These findings suggest that the DEGs involved in sepal morphogenesis regulation may also participate in cytokinesis.

KEGG pathway enrichment analysis revealed several significantly enriched pathways, including biosynthesis of secondary metabolites, fatty acid degradation, and starch and sucrose metabolism, all of which play important roles in plant development (**Supplementary Figure S2**; **Supplementary Table S8**). Metabolites from the phenylpropanoid biosynthesis pathway show organ-specific and developmental patterns (Vogt, 2010). Additionally, pathways related to fatty acid metabolism and amino acid metabolism, including tyrosine metabolism, cysteine and methionine metabolism, cyanoamino acid metabolism, and alpha-Linolenic acid metabolism, were significantly enriched in our data. These pathways and their derivatives are known to be involved in floral organ development (Chakraborty et al., 2022). Notably, the DEGs enriched in pathways such as carbon metabolism, fatty acid degradation, glyoxylate and dicarboxylate metabolism, and starch and sucrose metabolism may work together to regulate plant energy storage, which is crucial for cytokinesis.

### 3.6 Functional characterization of the key DEGs and their role in sepal curvature and morphogenesis

**Figure 7:**
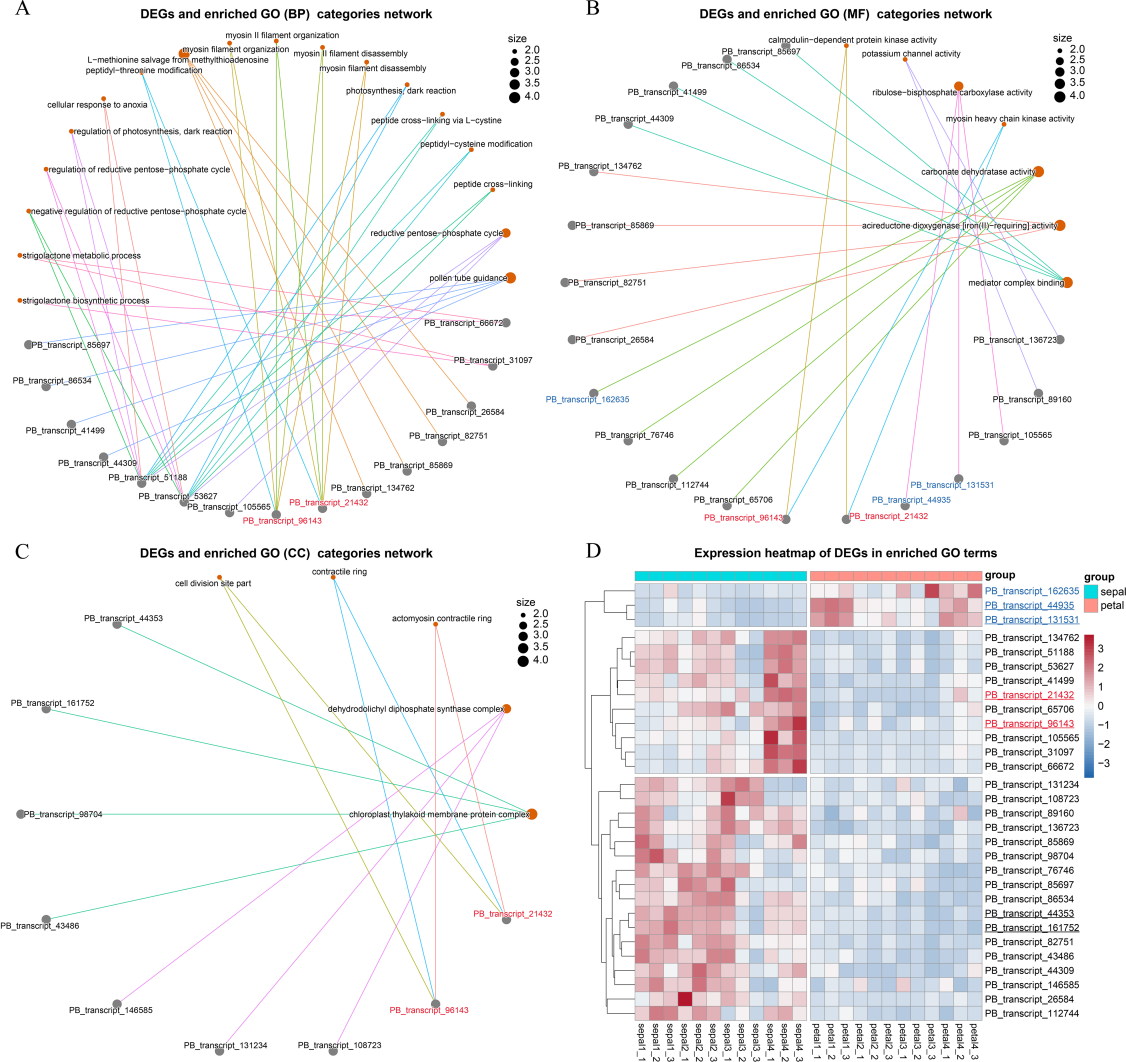
Network visualization of GO terms and their enriched genes. **(A)** Network illustrating the linkages between genes and enriched GO terms within the Biological Process (BP) category. Each node represents a GO term, and lines (edges) indicate shared genes enriched in multiple GO terms, forming interconnections between the terms and genes. **(B)** Network showing the linkages between genes and enriched GO terms within the Molecular Function (MF) category. **(C)** Network of genes and enriched GO terms within the Cellular Component (CC) category. **(D)** Stage-specific expression analysis of genes enriched in GO biological processes. The underlined transcript IDs were selected for the qRT-PCR validation experiment.

Genes can be annotated with multiple GO categories, and identifying key DEGs that are linked to several GO categories can provide deeper insights into their functional roles. To explore this, we constructed linkage network maps to illustrate the connections between the key GO terms and the associated 30 DEGs (**Figures 7A–C**). Among the 30 DEGs, 27 were upregulated, while only 3 were downregulated in sepals (**Figure 7D**). Notably, PB_transcript_51188 and PB_transcript_53627 were associated with diverse GO biological process (BP) categories, including the strigolactone biosynthetic process, reductive pentose-phosphate cycle, and peptide cross-linking (**Figure 7A**). These two DEGs also exhibited similar expression patterns, with high expression observed in Stage 1, Stage 2, and Stage 4 sepals (**Figure 7D**). Recent studies have shown that cross-linking proteins play a role in regulating actin filament network organization and force generation in yeast (Hill et al., 2024). These findings suggest that PB_transcript_51188 and PB_transcript_53627 may contribute to sepal morphogenesis, potentially by regulating the actin filament network.

In addition, two other DEGs, PB_transcript_96143 and PB_transcript_21432, were found to share the same BP, MF, and CC categories (**Figures 7A–C**). The proteins they encode possess calmodulin-dependent protein kinase activity and are involved in myosin II filament organization at the actomyosin contractile ring subcellular location. These two DEGs also showed high expression during sepal development, particularly at Stage 4 (**Figure 7D**). Calmodulin-dependent protein kinase regulates enzymatic activity and cytoskeletal organization by phosphorylating proteins such as myosin light chain kinase (Tokumitsu and Sakagami, 2022). During cytokinesis, the accumulation of actin and myosin II filaments at the cell equator forms a contractile ring that helps divide the cell into two daughter cells (Arima et al., 2023). These results further support the involvement of the key DEGs in the process of cytokinesis.

**Figure 8:**
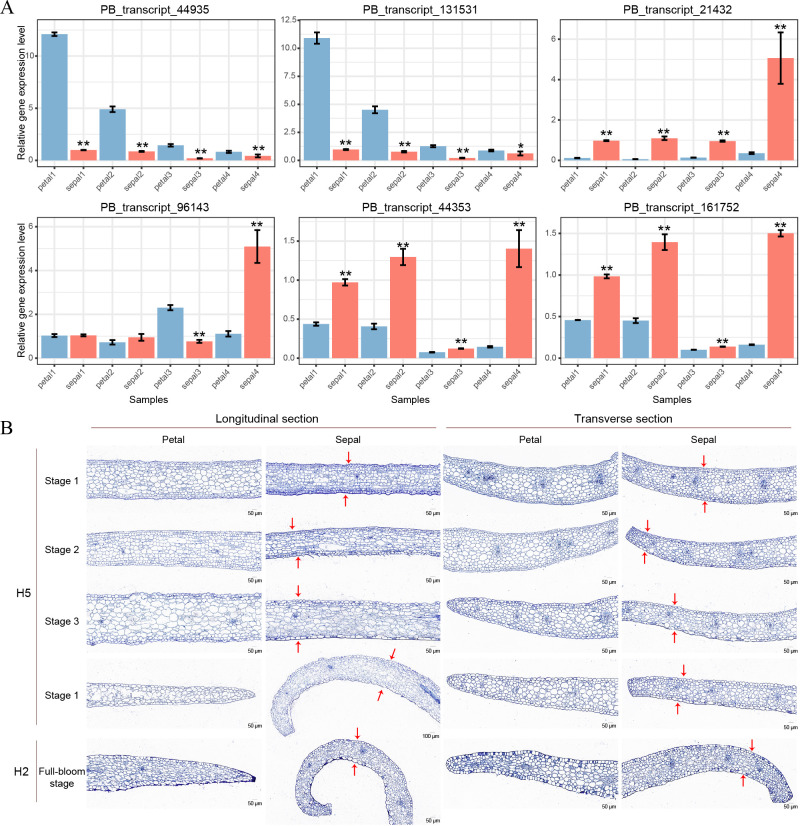
qRT-PCR validation and paraffin sections of petals and sepals in *Den.* Sect. *Spatulata* hybrids. **(A)** qRT-PCR validation of six selected genes across developmental Stages 1-4 in petals and sepals of *Den.* Sect. *Spatulata* hybrids. Statistical significance, determined by Student’s t-test, is indicated as follows: *P < 0.05; **P < 0.001. Number of replicates = 3. **(B)** Paraffin sections of petals and sepals from *Den. Sect. Spatulata* hybrids. Stages 1 to 4 display paraffin sections of petals and sepals from H5 at different developmental stages, while H2 represents sections of petals and sepals at the full-bloom stage. Red arrows above and below the sepals denote the key differences in cell number and size observed in this study.

To validate the RNA-Seq results, we conducted qRT-PCR analysis on six selected genes, with two genes representing each of the three clusters shown in **Figure 7D**. PB_transcript_44935 and PB_transcript_131531 showed consistent downregulation in sepals throughout all four developmental stages (**Figure 8A**). Conversely, PB_transcript_21432, PB_transcript_44935, and PB_transcript_161752 were significantly upregulated in sepals across all stages. Notably, PB_transcript_96143 displayed a unique expression pattern, with suppression at Stage 3 followed by marked activation at Stage 4 in sepals. Overall, these expression patterns align well with the RNA-Seq data (**Figure 7D**), indicating a high level of concordance between the qRT-PCR and RNA-Seq findings. This consistency supports the reliability of the expression patterns identified across these conditions. To reveal whether the cell number and cell size were different between sepals and petals, the tissue sections of sepals and petals were visualized and compared in **Figure 8B**. The cell number and size were approximately the same in the upper and bottom layers of the petals. By contrast, the cell number from the upper layers was much higher than those in the bottom layers of the sepals. Meanwhile, the cell size in the upper layers was much smaller than that in the lower layers (**Figure 8B**). A similar cell distribution pattern was also observed in H2 sepals, whose sepals are even more curled than H5 (**Figure 1A**). These results support the hypothesis that the curled sepals are possibly caused by the mechanical force from the increased number of cells.

## 4 Discussion

This study investigated the morphological and molecular mechanisms underlying sepal curvature in *Den*. Sect. *Spatulata* hybrids, focusing on hybrid H5, which exhibited pronounced sepal curling compared to its petals (**Figure 1**). The data collected, including transcriptomic profiles and histological analysis, revealed key cellular and molecular processes driving sepal curvature, notably involving differential cell growth, cytokinesis, and cytoskeletal organization.

Transcriptomic analysis revealed a significant number of DEGs between the sepals and petals, with 72.8% of these DEGs upregulated in sepals (**Figure 5**). Many of these genes are involved in processes related to cytokinesis and cytoskeletal dynamics, such as myosin II filament organization (**Figure 6**). Cytoskeletal reorganization is essential for morphogenesis, as it contributes to both cell division and mechanical force generation. This is consistent with studies that highlight the role of myosin II in cytokinesis, where myosin filaments assemble into contractile rings that facilitate membrane invagination and division (Najafabadi et al., 2022; Arima et al., 2023). Additionally, actin cross-linking proteins, such as α-actinin, regulate actin filament length and alignment, contributing to force generation during cellular processes (Hill et al., 2024). The observed upregulation of genes encoding actin-crosslinking proteins and myosin motors during sepal development suggests that these proteins coordinate to generate the mechanical forces required for sepal curling. This mechanical model aligns with previous studies showing that actin filament organization is tightly regulated by calcium-dependent actin-binding proteins, which contribute to cytoskeletal reorganization in response to intracellular signaling (Lehne and Bogdan, 2023; Rajan et al., 2023).

Interestingly, key DEGs involved in myosin activity were upregulated in sepals during the later stages of development (**Figure 7D**), suggesting a role for cytoskeletal dynamics in driving the more pronounced curvature observed at these stages. Previous studies have shown that myosin motors can act as mechanosensors, not only generating forces but also responding to mechanical stimuli in various cellular contexts (Greenberg et al., 2016; Pillon and Doublet, 2021). This dual role of myosins could explain how mechanical feedback loops, involving both force generation and mechanosensing, help shape the sepal. In particular, non-muscle myosin II (NMII) filaments have been shown to self-organize and contribute to the regulation of cytoskeletal contractility during cytokinesis (Najafabadi et al., 2022).

Metabolic pathways also appeared to be involved in sepal morphogenesis. Significant enrichment in pathways related to energy metabolism, such as fatty acid degradation and starch and sucrose metabolism (**Supplementary Figure S2**; **Supplementary Table S8**), likely provides the energy necessary for cytokinesis and other growth-related processes. These findings are consistent with recent studies demonstrating the interplay between energy metabolism and the cell cycle, where metabolic fluctuations synchronize with cell proliferation to support organ growth (Siqueira et al., 2018). The coordination of energy metabolism with cytoskeletal dynamics is likely crucial for supporting the high energy demands of cell division and tissue expansion in sepals.

In sepal morphogenesis, both cytokinesis and mechanical force generation play crucial yet distinct roles. Cytokinesis facilitates the increase in cell numbers by driving cell division, contributing to tissue growth (Glotzer, 2005). In our study, the cells in the upper layers of the sepal were observed to be smaller and more numerous than those in the lower layers (**Figure 8**), suggesting that cytokinesis plays a role in expanding cell populations. However, the upregulation of myosin II filament organization, a key player in generating mechanical forces along actin filaments, indicates that mechanical tension is the primary driver of sepal curvature (Martin et al., 2009; Vicente-Manzanares et al., 2009). While cytokinesis promotes cell proliferation, it does not directly account for the structural curvature. Instead, the contraction and organization of myosin II filaments, which generate forces across the cytoskeleton, likely cause the differential tension between cell layers, resulting in the observed curling of the sepals (Lecuit and Lenne, 2007). Interestingly, recent studies in *Arabidopsis* sepals have shown that variation in cell size modestly contributes to sepal width but does not affect the overall robustness of sepal shape, further supporting the idea that shape is governed by mechanical forces rather than just cell number or size variability (Trinh et al., 2024). Mechanical forces, particularly those generated by the cytoskeleton, have been shown to play a key role in plant tissue morphogenesis, as observed in other plant structures (Hamant et al., 2008). Thus, although cytokinesis contributes to the overall tissue architecture, the curvature of the sepal is predominantly shaped by the mechanical forces generated by myosin II activity.

While this study provides valuable insights into sepal curvature in *Den.* Sect*. Spatulata*, a key limitation is the absence of functional validation to confirm the roles of the identified genes. Future research should address this by utilizing advanced genetic transformation techniques, such as Agrobacterium-mediated transformation or CRISPR/Cas9, which have been successfully applied in *Dendrobium* species (Phlaetita et al., 2015; Kui et al., 2016). Gene knockdown or overexpression experiments could help validate the involvement of specific genes in processes such as actin organization and myosin activity, which are crucial for cytoskeletal dynamics and mechanical force generation in sepal morphogenesis (Greenberg et al., 2016). Such functional studies, combined with transcriptomic profiling, would provide direct evidence for the molecular pathways driving sepal curvature and offer potential for enhancing morphological traits in *Dendrobium* through genetic modification.

## 5 Conclusion

This study provides valuable insights into the morphological and molecular mechanisms underlying sepal curvature in *Den.* Sect. *Spatulata*, particularly in hybrid H5. Our results demonstrate that sepal curvature is primarily driven by mechanical forces generated by the actomyosin cytoskeleton, rather than by an increase in cell numbers alone. While differential cell growth, characterized by smaller and more numerous cells in the upper sepal layers, contributes to the overall tissue architecture, the upregulation of genes involved in actin filament organization and myosin motor activity suggests that mechanical tension plays a central role in shaping sepal curvature. Transcriptomic analysis identified key DEGs associated with cytokinesis, cytoskeletal organization, and energy metabolism, highlighting a coordinated network of mechanical and metabolic processes that regulate sepal morphogenesis. The involvement of myosin II filament organization in generating contractile forces further supports the hypothesis that mechanical signaling, rather than cell proliferation alone, is critical for the development of the distinctive sepal shape.

These findings advance our understanding of the molecular basis of sepal curvature and floral organ morphogenesis in *Dendrobium*, expanding upon existing models of organ development in plants. Our work also opens new avenues for exploring how mechanical forces, energy metabolism, and hormonal signaling interact to drive floral organ shape. By integrating transcriptomic data with histological observations, this study offers a comprehensive framework for investigating the molecular and cellular mechanisms that govern the development of complex floral structures in orchids, with broader implications for plant developmental biology and evolutionary studies.

## Data Availability

The data presented in the study are deposited in the NCBI Sequence Read Archive (SRA) repository, under BioProject accession number PRJNA1129577.
