# Supplementary material for: Unveiling the molecular mechanism of sepal curvature in Dendrobium Section Spatulata through full-length transcriptome and RNA-seq analysis
Source: Front Plant Sci. 2024 Dec 13;15:1497230. doi: 10.3389/fpls.2024.1497230 (PMC11671263; doi:10.3389/fpls.2024.1497230)
Supplement: Supplementary file 1 [file DataSheet1.pdf]

Supplementary Material

Supplementary Figures

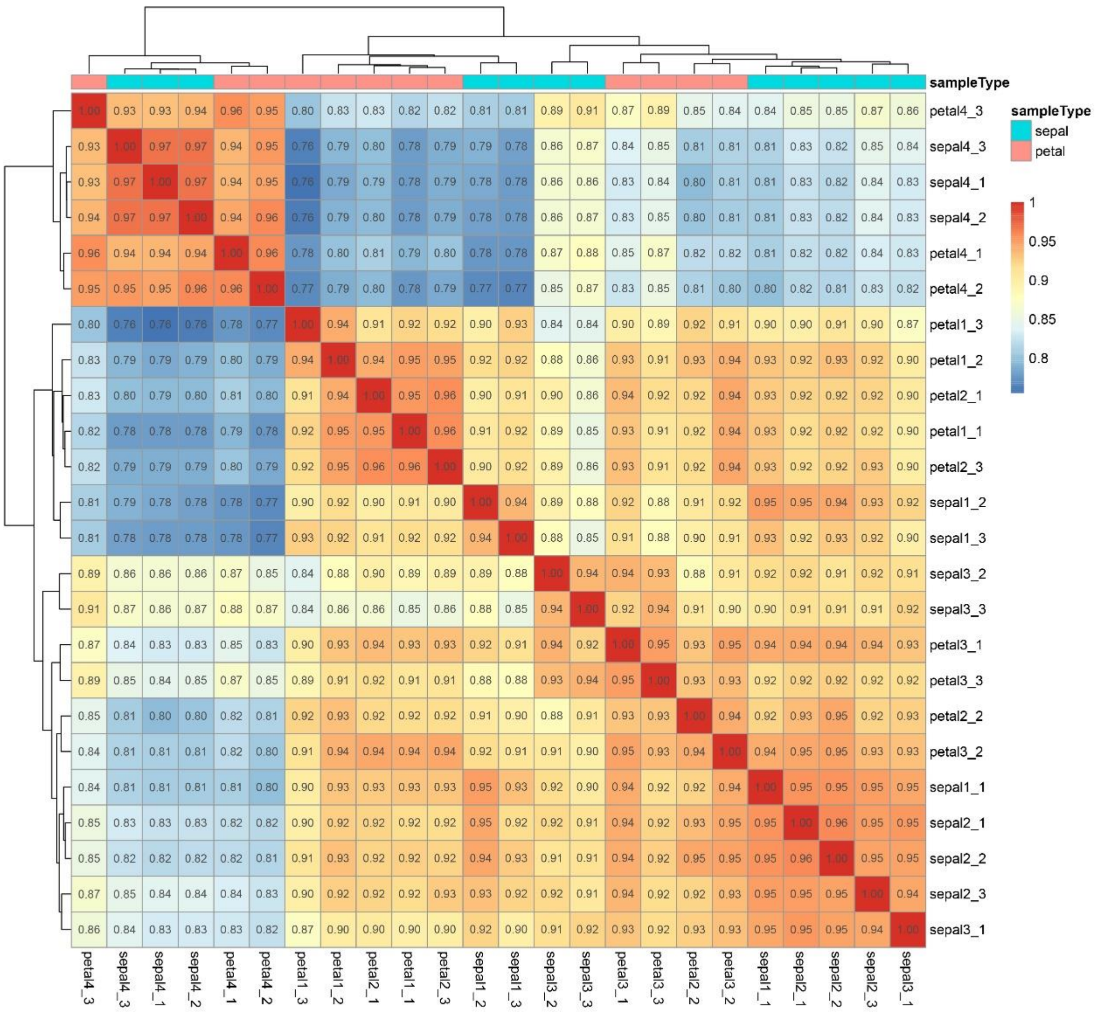

Supplementary Figure 1. Correlation of gene expression between RNA-seq samples.

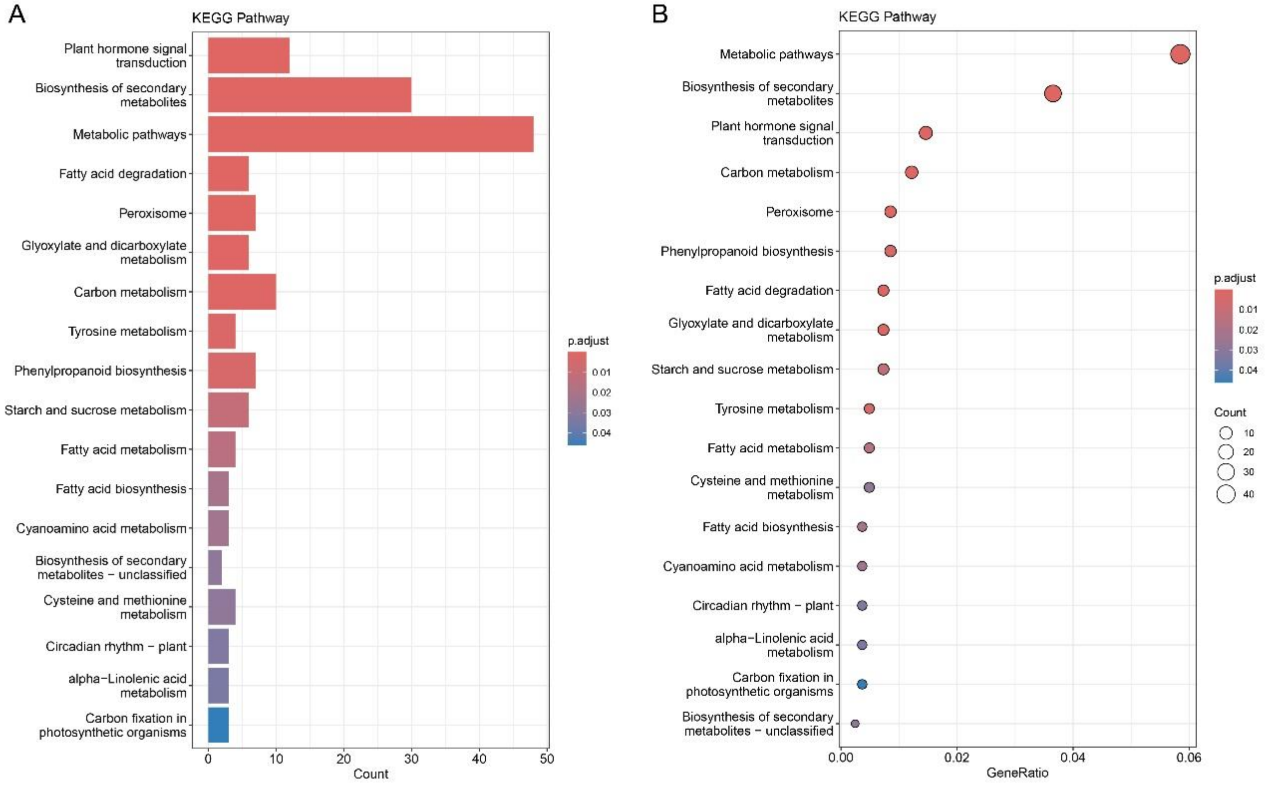

Supplementary Figure 2. KEGG pathway enrichment analysis of DEGs. (A) Bar plot of significantly enriched KEGG pathways for all DEGs. (B) Dot plot of significantly enriched KEGG pathways for all DEGs
